# Admission C-reactive protein concentrations are associated with unfavourable neurological outcome after out-of-hospital cardiac arrest

**DOI:** 10.1038/s41598-021-89681-8

**Published:** 2021-05-13

**Authors:** Christoph Schriefl, Christian Schoergenhofer, Michael Poppe, Christian Clodi, Matthias Mueller, Florian Ettl, Bernd Jilma, Juergen Grafeneder, Michael Schwameis, Heidrun Losert, Michael Holzer, Fritz Sterz, Andrea Zeiner-Schatzl

**Affiliations:** 1 Department of Emergency Medicine, Medical University of Vienna, Vienna, Austria; 2 Department of Clinical Pharmacology, Medical University of Vienna, Vienna, Austria

**Keywords:** Biomarkers, Outcomes research

## Abstract

Whether admission C-reactive protein (aCRP) concentrations are associated with neurological outcome after out-of-hospital cardiac arrest (OHCA) is controversial. Based on established kinetics of CRP, we hypothesized that aCRP may reflect the pre-arrest state of health and investigated associations with neurological outcome. Prospectively collected data from the Vienna Clinical Cardiac Arrest Registry of the Department of Emergency Medicine were analysed. Adults (≥ 18 years) who suffered a non-traumatic OHCA between January 2013 and December 2018 with return of spontaneous circulation, but without extracorporeal cardiopulmonary resuscitation therapy were eligible. The primary endpoint was a composite of unfavourable neurologic function or death (defined as Cerebral Performance Category 3–5) at 30 days. Associations of CRP levels drawn within 30 min of hospital admission were assessed using binary logistic regression. ACRP concentrations were overall low in our population (n = 832), but higher in the unfavourable outcome group [median: 0.44 (quartiles 0.15–1.44) mg/dL vs. 0.26 (0.11–0.62) mg/dL, *p* < 0.001]. The crude odds ratio for higher aCRP concentrations was 1.19 (95% CI 1.10–1.28, *p* < 0.001, per mg/dL) to have unfavourable neurological outcome. After multivariate adjustment for traditional prognostication markers the odds ratio of higher aCRP concentrations was 1.13 (95% CI 1.04–1.22, *p* = 0.002). Sensitivity of aCRP was low, but specificity for unfavourable neurological outcome was 90% for the cut-off at 1.5 mg/dL and 97.5% for 5 mg/dL CRP. In conclusion, high aCRP levels are associated with unfavourable neurological outcome at day 30 after OHCA.

## Introduction

Out-of-hospital cardiac arrest (OHCA) remains a major public health problem that claims over 770,000 lives in Europe and the US annually^1,2^. Whilst significant efforts have been made to increase layperson awareness and bystander cardiopulmonary resuscitation (CPR), only 10% of patients admitted after OHCA survive to hospital discharge, many of whom with permanent brain injury^2,3^.

The early phase after successful CPR with return of spontaneous circulation (ROSC) is characterized by global ischemia and reperfusion injury. The pathophysiological mechanisms in this period are summarized as “post cardiac arrest syndrome”, which features a systemic inflammatory response, hemodynamic instability and organ dysfunction^4,5^. The degree of the inflammatory response after CPR is associated with clinical outcomes^5,6^. However, whether inflammatory conditions that are present before cardiac arrest may impact on clinical outcomes remains to be established, which was the main focus of this analysis. Given the well-known kinetics of C-reactive protein^7^, measurements immediately after hospital admission reflect the pre-arrest state of health of patients and are unlikely to be relevantly influenced by the process of CPR itself, which is in contrast to blood samples drawn later during post-resuscitation care.

Earlier studies showed inconclusive results: Isenschmid et al. reported that higher C-reactive protein levels were associated with poor neurologic outcome, but neither specified the timing of blood sampling nor the exact population (OHCA or in-hospital cardiac arrest (IHCA))^8^. Dell’anna et al. reported similar results, but included both, OHCA and IHCA patients^9^. It needs to be noted that IHCA patients differ relevantly from patients with OHCA, because hospitalized patients almost regularly have increased biomarkers of systemic inflammation, e.g. due to surgery or infectious diseases, and the underlying causes of cardiac arrest differ accordingly^10,11^. In contrast, Vaahersalo et al. included only OHCA patients, but found no differences in C-reactive protein levels between patients with good or poor neurologic outcome. “Admission” blood sampling was only performed within 6 h of hospital admission^6^. Interestingly, interleukin-6 levels differed significantly between these two groups. Since C-reactive protein is a downstream marker of interleukin-6^12^ with well-known kinetics, these results emphasise the importance of timing of blood analysis.

Given these limitations and partly contradictory data, we analysed data from a large prospective single centre registry to determine potential associations between C-reactive protein levels measured immediately after hospital admission (within 30 min) and clinical outcomes after OHCA.

## Methods

### Study design

The current analysis is based on prospectively collected data from the Vienna Clinical Cardiac Arrest Registry of the Department of Emergency Medicine. The registry includes all adult cardiac arrest patients, admitted to, and treated at, the Department of Emergency Medicine of the Medical University of Vienna, a tertiary care facility.

Data acquisition and documentation was conducted in accordance with the Utstein style recommendations for cardiac arrest related documentation^13^. Data were obtained by a health care professional either via personal visits or via standardised telephone interviews if the patient had been discharged from hospital. Health care professionals were unaware of C-reactive protein levels during the assessments of neurologic function. Baseline data, for example witnessed status, were determined carefully through meticulous communication with the dispatch centre, the emergency physicians, the paramedics on scene, bystanders and relatives.

Blood samples were drawn immediately after hospital admission (at maximum within 30 min) and analysed by the ISO-certified central laboratory of the Vienna General Hospital. The first blood sample always includes C-reactive protein (mg/dL) as a standard marker of inflammation.

This study was approved by the local Ethics Committee of the Medical University of Vienna (EK No. 1219/2018) with waiver of informed consent due to the minimal risks arising from study participation. The study complies with the Declaration of Helsinki and was performed in accordance with the relevant guidelines and regulations.

### Study population

In our analysis cohort we included all adults ≥ 18 years of age who suffered a non-traumatic out of hospital cardiac arrest between January 2013 and December 2018. Patients without return of spontaneous circulation and those with extracorporeal cardiopulmonary resuscitation (eCPR) therapy were excluded. Furthermore, we excluded patients with missing data for the multivariate analysis.

Patients were treated by the local emergency medical service, operated by the Municipal Ambulance Service Vienna and supported by partner organizations, on scene. Emergency medical services (EMS) performed advanced life support on scene according to standard operating procedures and current guidelines. We have recently described the Viennese EMS in detail^14^. Patients were admitted to one of the intensive care positions at the Department of Emergency Medicine. Patient treatment was based on current guidelines, including post-resuscitation management^1,15^. Targeted temperature management (32–34 °C) was conducted for all comatose patients according to an institutional protocol based on the current guidelines for 24 h until the start of rewarming.

### Endpoints

The primary endpoint was neurological outcome at day 30, defined as follows: favourable neurological outcome was defined as a Cerebral Performance Category (CPC) 1 (good neurologic function) or 2 (moderate disability); unfavourable neurological outcome was defined as CPC 3–5 (severe disability, vegetative state, or death) or persistent unresponsiveness due to analgosedation during the study period or before death. This is in accordance with the Utstein recommendations^16^.

The secondary endpoint was 30-day mortality.

### Statistical analysis

We present categorised data as counts (relative frequency), and continuous data as median ± quartiles. C-reactive protein levels on admission were compared between patients with favourable and unfavourable neurological outcome at day 30 using the Mann–Whitney U test.

We used a binary logistic regression analysis (backward stepwise elimination approach according to Wald test-statistics step-by-step) to estimate the effect of admission C-reactive protein levels on the primary endpoint. The effect was quantified as odds ratio with 95% confidence intervals (95% CI).

We used a Cox regression analysis (backward stepwise elimination approach according to Wald test-statistics step-by-step) to estimate the effect of admission C-reactive protein levels on the secondary endpoint taking the time-to-event into account. The effect was quantified as hazard ratio with 95% CI.

We selected covariables for the multivariable models based on both clinical reasoning and previous studies. These variables included age, sex, initial shockable rhythm, basic life support, witness status, number of shocks, cumulative epinephrine dose, and pH. The final results of the multivariate analyses are presented in tables. Those variables eliminated by the testing procedure were omitted.

We conducted receiver-operating characteristic (ROC) curve analysis to assess the performance of C-reactive protein concentrations for outcome parameters (based on sensitivity and specificity) and to identify respective cut-offs. Moreover, the added value of C-reactive protein to (1) our model, (2) the TTM risk score^17^ and (3) the CAHP score^18^ was assessed by comparing derived areas under the ROC curve by the DeLong test.

We performed a sensitivity analysis for the primary and secondary endpoint in patients with presumed cardiac cause of cardiac arrest. Furthermore, we included dichotomized data (based on cut-offs) in the analysis instead of the continuous variable (Supplement). Kaplan–Meier Curve plots of the estimated 30-day survival were drawn for these cut-offs and groups were compared using the log-rank test.

For data management and analyses we used MS Excel (Microsoft Corporation Redmond, USA) and IBM SPSS Statistics (Version 26, IBM Corporation) as well as R (R Foundation for Statistical Computing, Version 3.6.2). A two-sided p-value < 0.05 was considered statistically significant.

## Results

**Figure 1:**
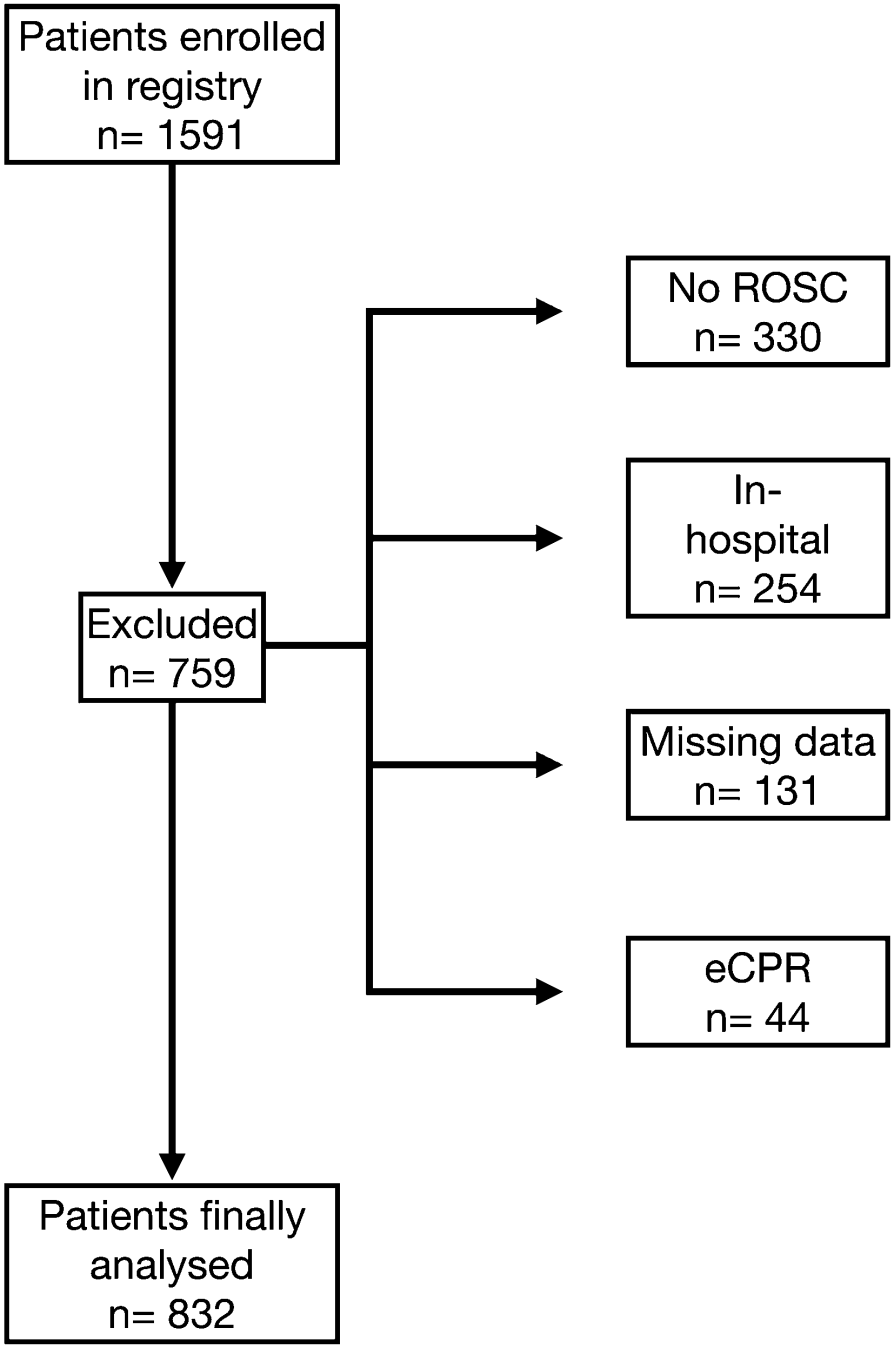
Flowchart of the study.

During the observation period 1591 cardiac arrest patients were enrolled in our registry. Of those patients, 832 patients fulfilled all inclusion criteria and were finally analysed (Fig. 1).

### Baseline characteristics

Table 1 shows baseline characteristics of the study patients, including distribution of cardiac arrest related parameters grouped by 30-day neurological outcome. In short, patients with unfavourable neurological outcome were older, less often had an initially shockable rhythm or a cardiac cause of cardiac arrest. They had higher blood lactate levels lower pH levels and overall more comorbidities. Interestingly, C-reactive protein levels were overall low with 490 of 832 patients having C-reactive protein levels within the reference range (0.5 mg/dL).

**Table 1:** Baseline characteristics by outcome group.

	All	Favourable neurological outcome	Unfavourable neurological outcome
	n = 832	n = 357	n = 475
Age, median (IQR)	60 (49–70)	55 (47–66)	63 (51–73)
Female, n (%)	236 (28)	92 (26)	144 (30)
Witnessed, n (%)	691 (83)	323 (90)	368 (78)
BLS, n (%)	505 (61)	239 (67)	266 (56)
**Initial rhythm, n (%)**			
VT	4 (1)	3 (0.8)	1 (0.2)
VF	440 (53)	257 (72)	183 (39)
PEA	191 (23)	50 (14)	141 (30)
Asystole	133 (16)	14 (4)	119 (25)
Unknown	63 (8)	32 (9)	31 (7)
No Flow (min), median (IQR)^a^	0 (0–1)	0 (0–1)	0 (0–1)
Low Flow (min), median (IQR)^a^	23 (14–37)	16 (10–25)	29 (20–48)
**Origin, n (%)**			
Pulmonary	114 (14)	26 (7)	88 (19)
Cardiac	547 (66)	284 (79)	263 (55)
Metabolic	11 (1)	4 (1)	7 (2)
Intoxication	25 (3)	5 (1)	20 (4)
Drowning	15 (2)	4 (1)	11 (2)
Sepsis	1 (0.1)	0 (0)	1 (0.2)
Other	18 (2)	3 (1)	15 (3)
Cerebral	34 (4)	5 (1)	29 (6)
Unknown	67 (8)	26 (7)	41 (9)
Total dose of epinephrine in mg, median (IQR)	2 (0–4)	0 (0–2)	3 (1–5)
Number of shocks applied, median (IQR)^b^	3 (1–6)	3 (1–5)	4 (2–7)
pH, median (IQR)	7.16 (7.02–7.27)	7.24 (7.12–7.30)	7.10 (6.95–7.31)
Lactate (mmol/l), median (IQR)	7.3 (4.3–10.8))	5.1 (3.0–8.1)	8.9 (6.2–12.1)
CRP (mg/dl), median (IQR)	0.33 (0.14–0.94)	0.26 (0.11–0.62)	0.44 (0.15–1.46)
**Chronic health conditions, n (%)**			
Diabetes	158 (19)	49 (14)	109 (23)
Hypertension	339 (41)	122 (34)	217 (46)
Current smoker	239 (29)	115 (32)	124 (26)
Chronic heart failure	89 (11)	31 (9)	58 (12)
Myocardial infarction	89 (11)	38 (11)	51 (11)
Cerebral vascular insufficiency	53 (6)	15 (4)	38 (8)
Coronary artery disease	163 (20)	66 (19)	97 (20)
Chronic obstructive pulmonary disease	105 (13)	34 (10)	71 (15)

Favourable neurological outcome was defined as cerebral performance category 1–2, Unfavourable neurological outcome as cerebral performance category 3–5.

*BLS* basic life support performed by bystanders, *CRP* c-reactive protein, *PEA* pulseless electrical activity, *IQR* inter quartile range, *VF* ventricular fibrillation, *VT* pulseless ventricular tachycardia.

^a^Data only for witnessed available.

^b^Data only from patients receiving at least one shock.

### Outcome analysis

C-reactive protein levels on admission were significantly higher in patients with unfavourable neurologic outcome (Table 1, Supplement/Figure S1, p < 0.001).

In univariate analysis, the crude odds ratio for unfavourable neurologic outcome was 1.19 (95% CI 1.10—1.28, *p* < 0.001) for C-reactive protein admission levels. After multivariate adjustment for age, sex, initial shockable rhythm, basic life support, witness status, number of shocks, cumulative epinephrine dose, and pH the adjusted odds ratio of C-reactive protein admission levels was 1.13 (95% CI 1.04–1.22, *p* = 0.003, Table 2).

**Table 2:** Multivariate analysis of the primary endpoint (n = 832).

Parameters	Unfavourable outcome (cerebral performance category 3–5)	
	CRP [mg/dL]	
	OR (95% CI)	p-value
CRP	1.13 (1.04–1.22)	0.003
Age	1.04 (1.03–1.05)	< 0.001
Male sex	0.72 (0.50–1.03)	0.07
Witnessed arrest	0.34 (0.22–0.55)	< 0.001
BLS	0.74 (0.54–1.03)	0.073
pH	0.01 (0.00–0.02)	< 0.001

Unfavourable outcome was analysed by binary logistic regression using a backward stepwise elimination approach according to Wald test statistic step-by-step. The presented parameters are the ones remaining in the final step of the model.

*BLS* basic life support, *CI* confidence interval, *CRP* C-reactive protein, *OR* odds ratio.

The overall proportion of 30-day mortality was 42% (n = 345). In univariate analysis, the crude hazard ratio of C-reactive protein levels for 30-day mortality was 1.07 (95% CI 1.05–1.10, *p* < 0.001). The estimates of C-reactive protein levels on 30-day mortality in the adjusted model showed a hazard ratio of 1.04 (95% CI 1.01–1.07, *p* = 0.01, Table 3).

**Table 3:** Multivariate analysis of the secondary endpoint (n = 832).

Parameters	30-day mortality	
	CRP [mg/dL]	
	HR (95% CI)	p-value
CRP	1.04 (1.01–1.07)	0.01
Age	1.03 (1.02–1.04)	< 0.001
BLS	0.70 (0.53–0.91)	0.007
pH Value	0.05 (0.03–0.11)	< 0.001

30-day mortality was assessed by the Cox regression model using a backward stepwise elimination approach according to Wald test statistic step-by-step. The presented parameters are the ones remaining in the final step of the model.

*BLS* basic life support, *CI* confidence interval, *CRP* C-reactive protein, *HR* hazard ratio.

In ROC analysis, C-reactive protein had an area under the curve of 0.60 (95% CI 0.56–0.64, *p* < 0.001) for neurologic outcome and similarly of 0.6 (95% CI 0.56–0.64, *p* < 0.001) for 30-day mortality. No obvious cut-offs were identifiable. Thus, we used a specificity-driven approach to identify cut-offs at 1.5 mg/dL (specificity 90% and sensitivity 24% for unfavourable 30-day neurologic outcome; specificity 89% and sensitivity 27% for 30-day mortality) and 5 mg/dL (specificity 97.5% and sensitivity 10% for unfavourable 30-day neurologic outcome; specificity 96% and sensitivity 10% for 30-day-mortality). The population was dichotomized using these cut-offs and these variables were included in logistic regression models. In short, the adjusted odds ratio of the cut-off at 1.5 mg/dL was 2.28 (95% CI 1.44–3.61, *p* < 0.001) to have poor neurologic outcome and 3.44 (95% CI 1.57–7.53, *p* = 0.002) for the cut-off at 5 mg/dL. Details are presented in the Supplement (Tables S1 and S2).

The added value of C-reactive protein to the model calculated with the above-mentioned variables, with the CAHP and the TTM risk score was assessed. Of note, C-reactive protein statistically improved the multivariate model of this study, as well as the CAHP model, although the improvement was not clinically meaningful (Supplement, Figure S2/S3/S4).

**Figure 2:**
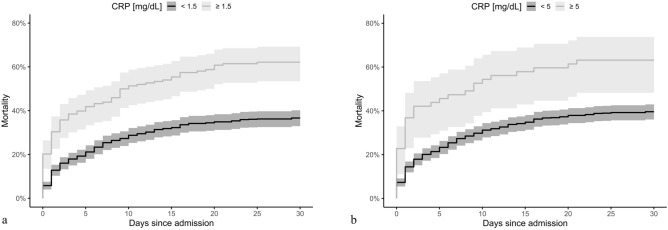
Kaplan–Meier plot of mortality to day 30 among ED patients presenting with OHCA according to (**a**) the CRP cut-off level of 1.5 mg/dL and (**b**) the CRP cut-off level of 5 mg/dL. Shaded areas indicate a 95% confidence interval. *CRP* C-reactive protein, *ED* emergency department, *OHCA* out-of-hospital cardiac arrest.

The Kaplan–Meier survival plot of the estimated 30-day mortality based on both cut-offs for C-reactive protein quartiles is presented in Fig. 2. The between-group difference continues throughout the observation time and is still observed on day thirty (log-rank test *p* < 0.001).

#### Patients with cardiac cause of OHCA

A total of 547 patients with an underlying cardiac cause of OHCA were included in this subgroup analysis. The crude OR for unfavourable neurological outcome was 1.54 (95% CI 1.26–1.88, *p* < 0.001) for C-reactive protein admission levels. After multivariate adjustment the adjusted OR was 1.45 (95% CI 1.20–1.75, *p* < 0.001, Table S3) for C-reactive protein.

The crude hazard ratio for 30-day mortality was 1.13 (95% CI 1.10–1.17, *p* < 0.001) for C-reactive protein admission levels. The estimates of C-reactive protein levels on 30-day mortality in the adjusted model showed a hazard ratio of 1.06 (95% CI 1.02–1.10, *p* = 0.005, Table S4) for C-reactive protein levels.

## Discussion

The main finding of this study is that admission C-reactive protein levels are associated with unfavourable neurological outcome and mortality 30-days after OHCA. Interestingly, the observed associations were even more pronounced in the subgroup of patients with an underlying cardiac cause of OHCA. C-reactive protein levels performed poorly in ROC analysis, which is explainable by a low sensitivity, although its specificity to predict unfavourable clinical outcomes was remarkable with a specificity of 90% at 1.5 mg/dL and 97.5% at 5 mg/dL.

In contrast to other studies^8,9^ we focused on OHCA patients in our analysis. As mentioned, IHCA and OHCA patients differ substantially^10,11^. Interestingly, Vaahersalo et al. reported that C-reactive protein levels did not differ between OHCA patients with favourable and unfavourable neurologic outcome. However, their sample size was much smaller (n = 130) compared to our study (n = 832) and their study may not have been sufficiently powered^6^.

The majority of studies in OHCA patients investigated parameters directly associated with CPR itself (e.g. serum lactate concentration or pH value)^19–23^. These parameters usually reflect no-flow time and low-flow time and the quality of resuscitation. Taking the kinetics of C-reactive protein into account^7^, admission C-reactive protein levels do not reflect the specifics of CPR, but rather the health status of patients prior to cardiac arrest. Although C-reactive protein synthesis starts rapidly after a single stimulus, it takes approximately 6 h for serum concentrations to rise above 0.5 mg/dL, and peak values are only reached after approximately 48 h^7^. Regrettably, the exact time of blood sampling and therefore the more relevant time span from cardiac arrest was not documented, but we can estimate this duration with sufficient accuracy for this analysis, as explained in the following. In contrast to other studies^6,8^, in our cohort blood samples were drawn shortly after admission (within 30 min). Schober et al. reported that the median time from the arrival of EMS at the scene of cardiac arrest to hospital admission was 50 (quartiles: 40–61) minutes in Vienna^24^. We included both, patients with witnessed and non-witnessed cardiac arrests. Thus, the time to arrival of EMS after CA cannot be exactly determined, however, the average time-to-first medical contact was 7 ± 3 min in Vienna^14^. In sum, blood samples were drawn within 60–90 min after cardiac arrest, which precludes substantial impact of cardiac arrest on C-reactive protein concentrations. In contrast, C-reactive protein levels measured after a time lag of six hours already may have been relevantly influenced by resuscitation associated aspiration, infection or inflammation, which could also explain negative results reported by Vahersalo et al.^6^. Likewise, Isenschmid et al. do not specifically state the timepoint of blood sampling in their study^8^.

C-reactive protein performed poorly in ROC curve analysis with an AUC of only 0.6. However, this is explainable by its low sensitivity. For instance, patients with low C-reactive protein levels may still experience unfavourable clinical outcomes, if their collapse is not witnessed, the no-flow time is exceedingly long or the quality of CPR is poor. Thus, the low sensitivity is not merely surprising, while the specificity is comparable to established risk scores and more established prognostic parameters: the specificity of the high-risk group score of the Cardiac Arrest Hospital Prognosis score was 98%, with a sensitivity of 36–51% (depending on the cohort)^18^, and the high-risk group in the target temperature risk (TTM) score: specificity 95%, sensitivity 40%^17^. Noteworthy, these scores only included witnessed patients, which contrasts our analysis. The specificity of neuron-specific enolase (NSE) was 94–100%, depending on the analysed cohorts, but only after 48 h, with a low sensitivity of 36%^25^. In sum, C-reactive protein concentrations measured immediately after hospital admission are not an optimal prognostic parameter given its low sensitivity, but the high specificity for poor neurologic outcome may indeed be of interest for clinicians, as it is a rapidly and ubiquitously available standard laboratory parameter measured.

The association of C-reactive protein concentrations with neurological outcome was more pronounced in the subgroup of patients with cardiac cause of cardiac arrest with an adjusted odds ratio of 1.45 (95% CI 1.20–1.75). C-reactive protein is recognised as a routine biomarker of cardiovascular disease^26–28^. Furthermore, there are numerous studies reporting associations of inflammation with poor clinical outcomes after myocardial infarction^29,30^.

The strengths of this analysis include the inclusion of a large cohort of patients, the prospective collection of data and the timely measurement of blood parameters shortly after admission. High quality ALS and post-resuscitation care are crucial determinants of patient outcomes^1^. We recently reported that EMS in the city of Vienna consistently demonstrate excellent cardiopulmonary resuscitation performance^14,31^. Moreover, the Department of Emergency Medicine is a specialized cardiac arrest center with high patient volumes^31^.

The major limitations of this study include its observational nature and its single centre design. Therefore, applying our results to other health care systems or countries may not be feasible. Other parameters of inflammation (e.g. interleukin-6) were not included in the analysis. We did not investigate reasons for high C-reactive protein levels in included patients, especially procalcitonin was not available for most patients. The exact time of blood sampling was not documented and consequently, the duration from cardiac arrest to blood sampling could only be estimated.

## Conclusions

In adult patients with OHCA high C-reactive protein levels on admission are associated with unfavourable neurological outcome and 30-day mortality. It is noteworthy that C-reactive protein levels do not reflect quality or duration of CPR, but rather the health status of patients prior to cardiac arrest.

## Supplementary Information

Supplementary Information.

## Data Availability

The datasets used and analysed during the current study are available from the corresponding author on reasonable request.

## References

[1] Soar J (2015). European resuscitation council guidelines for resuscitation 2015: Section 3 adult advanced life support. Resuscitation.

[2] Daya MR (2015). Out-of-hospital cardiac arrest survival improving over time: Results from the resuscitation outcomes consortium (ROC). Resuscitation.

[3] Young, G. B. Clinical practice. Neurologic prognosis after cardiac arrest. *N. Engl. J. Med.* **361**, 605–611. 10.1056/NEJMcp0903466 (2009). 10.1056/NEJMcp0903466 19657124

[4] Bro-Jeppesen, J. *et al.* The complement lectin pathway protein MAp19 and out-of-hospital cardiac arrest: Insights from two randomized clinical trials. *Eur. Heart J. Acute Cardiovasc. Care*, 2048872619870031. 10.1177/2048872619870031 (2019). 10.1177/2048872619870031 31538810

[5] Bro-Jeppesen J (2017). Level of systemic inflammation and endothelial injury is associated with cardiovascular dysfunction and vasopressor support in post-cardiac arrest patients. Resuscitation.

[6] Vaahersalo J (2014). Admission interleukin-6 is associated with post resuscitation organ dysfunction and predicts long-term neurological outcome after out-of-hospital ventricular fibrillation. Resuscitation.

[7] Pepys MB, Hirschfield GM (2003). C-reactive protein: A critical update. J. Clin. Invest..

[8] Isenschmid C (2018). Routine blood markers from different biological pathways improve early risk stratification in cardiac arrest patients: Results from the prospective, observational COMMUNICATE study. Resuscitation.

[9] Dell'anna AM (2014). C-reactive protein levels after cardiac arrest in patients treated with therapeutic hypothermia. Resuscitation.

[10] Fredriksson M (2010). Cardiac arrest outside and inside hospital in a community: Mechanisms behind the differences in outcome and outcome in relation to time of arrest. Am. Heart J..

[11] Herlitz J (2000). A comparison between patients suffering in-hospital and out-of-hospital cardiac arrest in terms of treatment and outcome. J. Intern. Med..

[12] Derhaschnig U (2004). Effect of interleukin-6 blockade on tissue factor-induced coagulation in human endotoxemia. Crit. Care Med..

[13] Perkins GD (2015). Cardiac Arrest and Cardiopulmonary Resuscitation Outcome Reports: Update of the Utstein Resuscitation Registry Templates for Out-of-Hospital Cardiac Arrest: A Statement for Healthcare Professionals From a Task Force of the International Liaison Committee on Resuscitation (American Heart Association, European Resuscitation Council, Australian and New Zealand Council on Resuscitation, Heart and Stroke Foundation of Canada, InterAmerican Heart Foundation, Resuscitation Council of Southern Africa, Resuscitation Council of Asia); and the American Heart Association Emergency Cardiovascular Care Committee and the Council on Cardiopulmonary, Critical Care Perioperative and Resuscitation. Resuscitation.

[14] Schriefl C (2019). Time of out-of-hospital cardiac arrest is not associated with outcome in a metropolitan area: A multicenter cohort study. Resuscitation.

[15] Nolan JP (2015). European Resuscitation Council and European Society of intensive care medicine guidelines for post-resuscitation care 2015: Section 5 of the European Resuscitation Council Guidelines for Resuscitation 2015. Resuscitation.

[16] Edgren E, Hedstrand U, Kelsey S, Sutton-Tyrrell K, Safar P (1994). Assessment of neurological prognosis in comatose survivors of cardiac arrest BRCT I Study Group. Lancet.

[17] Martinell L (2017). Early predictors of poor outcome after out-of-hospital cardiac arrest. Crit. Care.

[18] Maupain C (2016). The CAHP (Cardiac Arrest Hospital Prognosis) score: A tool for risk stratification after out-of-hospital cardiac arrest. Eur. Heart J..

[19] Carden DL, Martin GB, Nowak RM, Foreback CC, Tomlanovich MC (1987). Lactic acidosis during closed-chest CPR in dogs. Ann. Emerg. Med..

[20] Donnino MW (2014). Initial lactate and lactate change in post-cardiac arrest: A multicenter validation study. Crit. Care Med..

[21] Sheps DS (1979). Resting peripheral blood lactate elevation in survivors of prehospital cardiac arrest: Correlation with hemodynamic, electrophysiologic and oxyhemoglobin dissociation indexes. Am. J. Cardiol..

[22] Shinozaki K (2011). Blood ammonia and lactate levels on hospital arrival as a predictive biomarker in patients with out-of-hospital cardiac arrest. Resuscitation.

[23] Williams TA (2016). Use of serum lactate levels to predict survival for patients with out-of-hospital cardiac arrest: A cohort study. Emerg. Med. Australas..

[24] Schober A (2016). Admission of out-of-hospital cardiac arrest victims to a high volume cardiac arrest center is linked to improved outcome. Resuscitation.

[25] Schrage B (2019). Neuron-specific-enolase as a predictor of the neurologic outcome after cardiopulmonary resuscitation in patients on ECMO. Resuscitation.

[26] an individual participant meta-analysis (2010). Emerging Risk Factors, C. *et al.* C-reactive protein concentration and risk of coronary heart disease, stroke, and mortality. Lancet.

[27] Hage FG (2014). C-reactive protein and hypertension. J. Hum. Hypertens..

[28] Hage FG, Szalai AJ (2009). The role of C-reactive protein polymorphisms in inflammation and cardiovascular risk. Curr. Atheroscler. Rep..

[29] Shimizu T (2019). Clinical significance of high-sensitivity C-reactive protein in patients with preserved renal function following percutaneous coronary intervention. Int. Heart J..

[30] Carrero JJ, Andersson Franko M, Obergfell A, Gabrielsen A, Jernberg T (2019). hsCRP level and the risk of death or recurrent cardiovascular events in patients with myocardial infarction: A healthcare-based study. J. Am. Heart Assoc..

[31] Uray T (2015). Quality of post arrest care does not differ by time of day at a specialized resuscitation center. Medicine.

