# Author Correction: Admission C-reactive protein concentrations are associated with unfavourable neurological outcome after out-of-hospital cardiac arrest

**DOI:** 10.1038/s41598-021-98687-1

**Published:** 2021-09-17

**Authors:** Christoph Schriefl, Christian Schoergenhofer, Michael Poppe, Christian Clodi, Matthias Mueller, Florian Ettl, Bernd Jilma, Juergen Grafeneder, Michael Schwameis, Heidrun Losert, Michael Holzer, Fritz Sterz, Andrea Zeiner‑Schatzl

**Affiliations:** 1 Department of Emergency Medicine, Medical University of Vienna, Vienna, Austria; 2 Department of Clinical Pharmacology, Medical University of Vienna, Vienna, Austria

Correction to: *Scientific Reports* 10.1038/s41598-021-89681-8, published online 13 May 2021

The original version of this Article contained errors in the Abstract.

“Adults (≥ 18 years) who suffered a non-traumatic OHCA between January 2013 and December 2018, without return of spontaneous circulation or extracorporeal cardiopulmonary resuscitation therapy were eligible.”

now reads:

“Adults (≥ 18 years) who suffered a non-traumatic OHCA between January 2013 and December 2018 with return of spontaneous circulation, but without extracorporeal cardiopulmonary resuscitation therapy were eligible.”

The original Article has been corrected.

