# Supplementary material for: Admission C-reactive protein concentrations are associated with unfavourable neurological outcome after out-of-hospital cardiac arrest
Source: Sci Rep. 2021 May 13;11:10279. doi: 10.1038/s41598-021-89681-8 (PMC8119412; doi:10.1038/s41598-021-89681-8)
Supplement: Supplementary file 1 — Supplementary Information. [file 41598_2021_89681_MOESM1_ESM.docx]

**Supplementary Information:
Admission C-reactive protein concentrations are associated with unfavourable neurological outcome after out of hospital cardiac arrest**

Christoph Schriefl, Christian Schoergenhofer, Michael Poppe, Christian Clodi, Matthias Mueller, Florian Ettl, Bernd Jilma, Juergen Grafeneder, Michael Schwameis, Heidrun Losert, Michael Holzer, Fritz Sterz, Andrea Zeiner-Schatzl.

**Table S1.** Multivariate Analysis of the primary endpoint with a CRP cut-off level at 1.5mg/dL (n=832)

Unfavourable outcome was analysed by binary logistic regression using a backward stepwise elimination approach according to Wald test statistic step-by-step. Instead of the continuous CRP (Results, Table 2) a cut-off level at 1.5 mg/dL was used. The presented parameters are the ones remaining in the final step of the model.

|  | **Unfavourable outcome (Cerebral Performance Category 3-5)** | |
| --- | --- | --- |
|  | **CRP [cut-off 1.5 mg/dL]** | |
| Parameters | **OR (95% CI)** | p-value |
|  |  |  |
| CRP | 2.28 (1.44-3.61) | <0.001 |
| Age | 1.04 (1.03-1.05) | <0.001 |
| Male Sex | 0.72 (0.51-1.03) | 0.072 |
| Witnessed arrest | 0.35 (0.22-0.55) | <0.001 |
| BLS | 0.77 (0.56-1.07) | 0.117 |
| pH | 0.006 (0.002-0.017) | <0.001 |
|  |  |  |

Abbreviations: BLS: basic life support; CI: confidence interval; CRP: C-reactive protein; OR: odds ratio

**Table S2.** Multivariate Analysis of the primary endpoint with a CRP cut-off level at 5 mg/dL (n=832)

Unfavourable outcome was analysed by binary logistic regression using a backward stepwise elimination approach according to Wald test statistic step-by-step. Instead of the continuous CRP (Results, Table 2) a cut-off level at 5 mg/dL was used. The presented parameters are the ones remaining in the final step of the model.

|  | **Unfavourable outcome (Cerebral Performance Category 3-5)** | |
| --- | --- | --- |
|  | **CRP [cut-off 5 mg/dL]** | |
| Parameters | **OR (95% CI)** | p-value |
|  |  |  |
| CRP | 3.44 (1.57-7.53) | 0.002 |
| Age | 1.04 (1.03-1.05) | <0.001 |
| Male Sex | 0.71 (0.50-1.02) | 0.063 |
| Witnessed arrest | 0.34 (0.22-0.55) | <0.001 |
| BLS | 0.72 (0.52-0.99) | 0.045 |
| pH | 0.006 (0.002-0.017) | <0.001 |
|  |  |  |

Abbreviations: BLS: basic life support; CI: confidence interval; CRP: C-reactive protein; OR: odds ratio

**Table S3.** Patients with cardiac cause of out-of-hospital cardiac arrest (n=547)

Unfavourable outcome was analysed by binary logistic regression using a backward stepwise elimination approach according to Wald test statistic step-by-step. The presented parameters are the ones remaining in the final step of the model.

|  | **Unfavourable outcome (Cerebral Performance Category 3-5)** | |
| --- | --- | --- |
|  | **CRP [mg/dL]** | |
| Parameters | **OR (95% CI)** | p-value |
|  |  |  |
| CRP | 1.45 (1.20-1.75) | <0.001 |
| Age | 1.05 (1.04-1.07) | <0.001 |
| Male Sex | 0.59 (0.36-0.95) | 0.031 |
| BLS | 0.55 (0.36-0.84) | 0.005 |
| pH | 0.003 (0.001-0.011) | <0.001 |
|  |  |  |

Abbreviations: BLS: basic life support; CI: confidence interval; CRP: C-reactive protein; OR: odds ratio

**Table S4.** Patients with cardiac cause of out-of-hospital cardiac arrest (n=547)

30-day mortality was assessed by the Cox regression model using a backward stepwise elimination approach according to Wald test statistic step-by-step. The presented parameters are the ones remaining in the final step of the model.

|  | **30-day mortality** | |
| --- | --- | --- |
|  | **CRP [mg/dL]** | |
| Parameters | **HR (95% CI)** | p-value |
|  |  |  |
| CRP | 1.06 (1.02-1.10) | 0.005 |
| Age | 1.05 (1.03-1.06) | <0.001 |
| BLS | 0.65 (0.48-0.87) | 0.004 |
| pH | 0.02 (0.01-0.05) | <0.001 |
|  |  |  |

Abbreviations: BLS: basic life support; CI: confidence interval; CRP: C-reactive protein; HR: hazard ratio

**Figure S1**. Boxplot of the CRP level (logarithmic scale) between patients with CPC score 1-2 and CPC score 3-5. (n=832)

Abbreviations: CRP: C-reactive protein; CPC: Cerebral Performance Category

**Figure S2.** Receiver Operating Characteristic (ROC) curves of three models to predict the 30-day mortality (n=556)

Model 1 (red) CAHP, AUC 0.825, 95% CI: 0.7914-0.8593 (DeLong)
Model 2 (blue). CRP added to the CAHP model, AUC 0.831, 95% CI: 0.798-0.8645 (DeLong)
Model 3 (green). Model derived from our data (including CRP, age, sex, witness status, basic life support, pH). AUC 0.750, 95% CI: 0.7092-0.7901 (DeLong)

Abbreviations: CAHP: Cardiac Arrest Hospital Prognosis; CRP: C-reactive protein

**Figure S3.** Receiver Operating Characteristic (ROC) curves of three models to predict the 30-day mortality (n=556)

Model 1 (red) TTM, AUC 0.840, 95% CI: 0.8079-0.8727 (DeLong)
Model 2 (blue). CRP added to the TTM model, AUC 0.843, 95% CI: 0.8105-0.8749 (DeLong)
Model 3 (green). Model derived from our data (including CRP, age, sex, witness status, basic life support, pH). AUC 0.750, 95% CI: 0.7092-0.7901 (DeLong)

Abbreviations: CAHP: Cardiac Arrest Hospital Prognosis; CRP: C-reactive protein

**Figure S4.** Receiver Operating Characteristic (ROC) curves of two models to predict the 30-day mortality (n=832)

Model 1 (red) Model derived from our data (including CRP, age, sex, witness status, basic life support, pH). AUC 0.784, 95% CI: 0.7524-0.8147 (DeLong)
Model 2 (blue). Model derived from our data (age, sex, witness status, basic life support, pH). AUC 0.774, 95% CI: 0.7429-0.8061 (DeLong)

Abbreviations: CRP: C-reactive protein
